# TRIM5 Acts as More Than a Retroviral Restriction Factor

**DOI:** 10.3390/v3071204

**Published:** 2011-07-15

**Authors:** Suresh de Silva, Li Wu

**Affiliations:** Center for Retrovirus Research, Department of Veterinary Biosciences, The Ohio State University, 1900 Coffey Road, Columbus, OH 43210, USA; E-Mail: suresh.de-silva@cvm.osu.edu

**Keywords:** TRIM5, retroviruses, capsid, innate immunity

## Abstract

The retrovirus restriction factor TRIM5α blocks post-entry infection of retroviruses in a species-specific manner. As a cellular E3 ubiquitin ligase, TRIM5α binds to the retroviral capsid lattice in the cytoplasm of an infected cell and accelerates the uncoating process of retroviral capsid, thus providing a potent restriction to HIV-1 and other retrovirus infections. The precise mechanism by which this restriction is imposed remains under scrutiny, and evidence is lacking to link the E3 ubiquitin ligase activity of TRIM5α to its ability to restrict retrovirus infection. In a recent study, Pertel and colleagues have uncovered the link between the two, providing compelling evidence to suggest that following the interaction with the retroviral capsid, TRIM5 triggers an antiviral innate immune response by functioning as a pattern recognition receptor [1]. This unique function of TRIM5 is dependent on its association with the E2 ubiquitin-conjugating enzyme complex UBC13-UEV1A and subsequent activation of the TAK1 kinase complex and downstream genes involved in innate immune responses. These findings have defined a novel function for TRIM5 as a pattern recognition receptor in innate immune recognition and provided valuable mechanistic insight into its role as a retroviral restriction factor. Here we discuss the significance of these new findings in understanding TRIM5-mediated HIV restriction.

Given their limited genomic capacity, retroviruses such as HIV efficiently hijack the target cell’s machinery to complete their lifecycle and generate virus progeny. Under the selective pressure of pathogenic infections, the host has evolved a variety of restriction factors to block retroviral replication, and consequently retroviruses have evolved viral antagonists to overcome these restrictions [2]. In the case of HIV, three major restriction factors with distinct modes of action have been identified, including APOBEC3G (apolipoprotein B mRNA-editing, enzyme-catalytic, polypeptide-like 3G) [3], tripartite motif-containing protein 5α (TRIM5α) [4], and tetherin (also known as BST-2 or CD317) [5,6]. However, HIV type 1 (HIV-1) possesses its own countermeasures in the likes of accessory proteins to engage and counteract these restriction factors. For instance, HIV-1 Vif protein counteracts APOBEC3G and HIV-1 Vpu protein counteracts tetherin. By contrast, no retroviral antagonist has been reported to overcome TRIM5α and the precise mechanism by which TRIM5α restricts HIV-1 replication in non-human primates remains to be defined (reviewed in [7,8]).

In 2004, Stremlau and colleagues identified rhesus macaque TRIM5α as the factor responsible for blocking HIV-1 infection in Old World monkeys [4]. By contrast, human TRIM5α does not efficiently block HIV-1 infection [4], indicating the species-specific nature of the restrictive capacity of TRIM5α. This initial study showed the retrovirus capsid to be the target of TRIM5α, and subsequent comparative studies with retroviruses from different genera have confirmed that TRIM5α-mediated restriction is dependent on its avidity for retroviral capsid [9]. Upon release into the target cell cytoplasm, the retroviral capsid is engaged by TRIM5α and forms an elaborate hexagonal lattice on top of the capsid and accelerates capsid uncoating, which blocks retrovirus infection [10–13]. However, cyclophilin A (CypA), another cytoplasmic protein in human cells, binds the HIV-1 capsid and protects HIV-1 from a putative CypA-regulated antiviral activity in human cells [14]. Interestingly, a previous study by the Luban laboratory uncovered a unique retrotransposition event in owl monkey, the only New World primate that is resistant to HIV-1, resulting in the expression of a TRIM5-Cyclophilin A (TRIMCyp) fusion protein that is potent in blocking HIV-1 infection [15]. The TRIMCyp fusion protein has now been successfully used in *in vitro* assays by Pertel and colleagues to delineate the restrictive mechanism of TRIM5α linking to innate immune signaling pathways [1].

In an effort to understand the mechanism by which TRIM5α restricts retrovirus infection, Pertel and colleagues investigated the potential of TRIM5α as a signal transducer capable of activating NF-κB and AP-1-responsive genes involved in innate immune response [1], which can induce an antiviral state in the infected cell. This rationale was based on recent reports suggesting that TRIM5 plays a role in signal transduction processes [16,17]. Pertel *et al*. demonstrated that human TRIM5α, which does not efficiently restrict HIV-1 infection, is capable of activating mitogen activated protein kinases (MAPK)- and NF-κB-dependent inflammatory genes, similar to those activated by the gram-negative bacterial cell wall component, lipopolysaccharide (LPS). Interestingly, the authors showed that TRIM5α knockdown in myeloid cells attenuates the expression of LPS-induced inflammatory genes and also rescues the infection of HIV-1, SIV and non-retroviruses (vesicular stomatitis virus and Newcastle disease virus) from an antiviral state established by LPS. These results suggest an involvement of TRIM5α in innate immune signaling, while the rescue of viral infection from LPS is independent of viral capsid recognition by TRIM5α. Further investigation revealed that TRIM5α interacts with transforming growth factor beta-activated kinase 1 (TAK1), TAK1-binding protein 2 (TAB2), and TAB3, components of the TAK1 kinase complex in order to activate MAPK and NF-κB signaling pathways. This association is also essential for the retrovirus capsid-specific restriction mediated by TRIM5α. The authors then asked the critical question whether TRIM5α’s E3 ubiquitin (Ub) ligase activity plays a role in its apparent innate immune signaling function.

Ubiquitination is a post-translational modification of proteins that involves the covalent attachment of Ub to one or more lysine residues of a cellular protein via the concerted activity of three classes of enzyme termed E1 (Ub-activating enzymes), E2 (Ub-conjugating enzymes), and E3 (Ub-ligases). Traditionally, ubiquitination has been synonymous with tagging proteins for proteasomal degradation; however, an important role of ubiquitination in regulating immune responses is now being unraveled and appreciated (reviewed in [18]). In an attempt to uncover the link between TRIM5α’s E3 ligase activity and retrovirus restriction, Pertel and co-workers revealed that TRIM5α in partnership with the heterodimeric E2 Ub-conjugating enzyme complex, UBC13-UEV1A (also known as UBE2N-UBE2V1), is capable of generating unattached, lysine 63 (K63)-linked Ub chains. Upon multimerization, these free polyubiquitin chains can activate the TAK1 kinase complex by autophosphorylation with the help of regulatory proteins, TAB2 and TAB3. These reactions were elegantly demonstrated *in vitro* by using the owl monkey TRIMCyp fusion protein to circumvent the technical challenges in purifying full-length recombinant TRIM5α protein. Moreover, human TRIM5α expressed and enriched from transfected human 293T cells also possesses the capacity to generate free K63 poly-Ub chains *in vitro* [1].

So how does TRIM5α trigger the formation of free K63-linked Ub chains and trigger an innate immune response during retrovirus infection? The authors suggest that TRIM5 does so by acting as a *bona fide* pattern recognition receptor that specifically senses retroviral capsid, which then triggers the E3 ligase activity of TRIM5 to activate the TAK1 kinase complex, resulting in the activation of innate immune signaling pathways. Furthermore, UBC13 and UEV1A, the components of the E2 Ub-conjugating enzyme complex, interact with TRIM5 in addition to their previously known cellular partner TRAF6 (tumor necrosis factor receptor associated factor 6), which is an E3 Ub-ligase and plays an important role in NF-κB regulatory pathways in the innate immune responses against pathogens [18]. Therefore, the authors concluded that the E3 Ub-ligase activity and inflammatory gene induction function of TRIM5 are linked through its interactions with cellular factors including UBC13, UEV1A and TAK1, which also enhance capsid-specific restriction activity of TRIM5 (Figure 1). The authors also suggest that the original function of TRIM5 was likely to act as a pattern recognition receptor rather than a retrovirus restriction factor [1]. In contrast to these *in vitro* findings, Perez-Caballero and colleagues have previously shown that TRIM5-CypA-mediated restriction of HIV-1 is independent of the ubiquitination and proteasome function in owl monkey kidney cells [19]. It remains unclear to what extent TRIM5-induced innate immune responses rely upon ubiquitination to restrict HIV-1 or other retroviruses in human cells. Given that TRIM5α blocks retroviral infection in a species-specific manner, it is unclear whether the TRIM5 function as an innate immune sensor is dependent on the specific interaction between TRIM5 and HIV-1 capsid protein. It would be interesting to investigate whether TRIM5 interactions with other viral capsid proteins can also trigger the innate immune responses.

Based on the study by Pertel *et al.*, it is now clear that TRIM5α’s dual role in retrovirus infection serves not only to restrict the virus by attacking the retroviral capsid core, but also concomitantly triggers an ‘alarm’ in the form of an innate immune response in the infected cell. As a result, TRIM-mediated innate immune response could possibly establish an antiviral state in neighboring uninfected cells, which would serve to reduce the spread of certain retroviruses. It is possible that these two processes mediated by TRIM5α are coupled; however, it appears that human TRIM5-mediated innate immune response to HIV-1 capsid cannot efficiently control HIV-1 infection in humans given the existing AIDS pandemic. It might be possible that human TRIM5α plays a role in LPS-triggered immune activation through Toll-like receptor 4-mediated signaling, which has been linked to the progression of chronic HIV-1 infection [20]. Perhaps HIV-1 has evolved other yet unknown mechanisms to escape TRIM5-mediated innate immune response in humans. For example, recent studies indicate that post-entry HIV-1 infection in human monocyte-derived dendritic cells and macrophages is blocked by a host protein named SAMHD1 [21,22]. This myeloid-cell-specific HIV-1 restriction may allow HIV-1 to avoid antiviral innate immune responses derived from dendritic cells, a group of important antigen-presenting cells that bridge the innate and adaptive immunity [23]. Further investigation into the function and the mechanism of human TRIM5α as an innate immune sensor for retrovirus capsid might shed light to developing more effective interventions against HIV-1 infection.

## Figures and Tables

**Figure 1:**
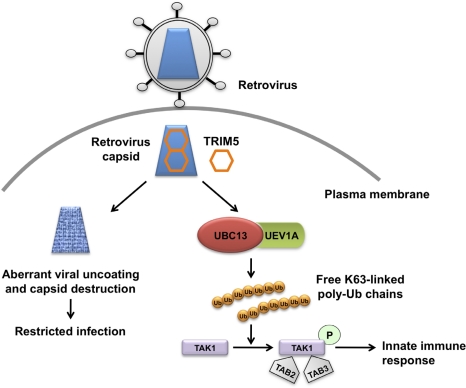
The dual role of TRIM5α in retrovirus infection. The retrovirus capsid is engaged by TRIM5α in the cytoplasm of the infected cell and forms a hexagonal lattice on top of the capsid. This leads to aberrant uncoating of the capsid and blocks retrovirus infection. Concomitantly, TRIM5α binding to capsid triggers its E3 ligase activity, and in concert with the E2 ubiquitin (Ub)-conjugating enzyme complex UBC13–UEV1A generates free lysine 63 (K63)-linked Ub chains, which in turn are catalysts in the autophosphorylation (indicated as a letter P in the green circle) of the TAK1 complex (includes TAK1, TAB2, and TAB3). Activation of the TAK1 complex by autophosphorylation results in the induction and expression of NF-κB- and MAPK-responsive inflammatory genes, thereby leading to an innate immune response in the infected cell.
